# Ferrite chitosan curcumin nanoparticles alleviate nandrolone decanote induced liver toxicity in male albino rats

**DOI:** 10.1038/s41598-025-21814-9

**Published:** 2025-10-29

**Authors:** Ehab Tousson, Afaf El Atrash, Somia Zaki, Marwa Negm, Amina I. Ghoneim

**Affiliations:** 1 Zoology Department, Faculty of Science, Tanta University, Tanta, Egypt; 2 Physics Department, Faculty of Science, Tanta University, Tanta, Egypt

**Keywords:** Nandrolone decanote, Nano ferrite-chitosan-curcumin nanoparticles, Liver, Oxidative stress, PCNA, TNFα, Biochemistry, Cell biology

## Abstract

Sr_2_NiCoAl_0.3_Fe_27.7_O_46_ X-Type hexa-nano-ferrites were prepared using a co-precipitation route, coated with Chitosan, and synthesized into a ferrite chitosan curcumin nanoparticle system. This combination enhances curcumin’s therapeutic properties, chitosan’s bioavailability, and ferrite’s magnetic targeting potential, offering improved cellular uptake and diagnostic applications. Nandrolone decanote (ND), one of the class II anabolic androgenic steroids (AASs), quickly spreading as a class of medications used in both clinical and illegal settings. AAS can enhance tolerance to exercise, so they are taken illegally by bodybuilders and adolescents. However, the abuse of AASs can lead to serious and irreversible organ toxicity. This study designed to investigate the therapeutics effect of nano ferrite chitosan curcumin nanoparticles (NF-CH-CurNPs) against ND induced hepatic toxicity, proliferation, inflammation, oxidative stress, and DNA damage in male rats. 48 adult male rats assigned to 6 groups [1st Gp, control; 2nd Gp, CurNPs; 3rd Gp, NF-CH-CurNPs; 4th Gp, ND; 5th Gp, ND + CurNPs; 6th Gp. ND + NF-CH-CurNPs]. Current results revealed, a significant elevation in the levels of aspartate transaminase (AST), alanine transaminase (ALT), total proteins, liver DNA damage, injury, malondialdehyde (MDA), proliferating cell nuclear antigen (PCNA), tumor necrosis factor alpha (TNFα) and a significant depletion in albumin, catalase, superoxide dismutase (SOD), reduced glutathione (GSH) after ND administration as compared to control and CurNPs groups. Treatment of ND with CurNPs or NF-CH-CurNPs significantly improved liver functions, structure, oxidative stress, DNA damage, PCNA, and TNFα expressions, with NF-CH-CurNPs showing potential for scavenging free radicals.

**Supplementary Information:**

The online version contains supplementary material available at 10.1038/s41598-025-21814-9.

## Introduction

Anabolic-androgenic steroids (AASs) are steroidal androgens, including natural androgens like testosterone, which have become a major public health concern due to their use in sports and other populations^1–4^. They are prescribed for treating hormonal imbalances, breast cancer, endometriosis, osteoporosis, and muscle loss. Misuse is common in athletes and non-athletes to increase body weight and lean muscle mass^5–7^. The lifetime prevalence of AASs is estimated to be 1 to 5%. The 2004 Anabolic Steroid Control Act redefined AAS to include drugs related to testosterone, including tetrahydrogestrinone and androstenedione^8,9^. Reduced fertility, high blood pressure, heart problems, atherosclerosis, tendon damage, and mental and behavioural issues are among the frequent side effects^10–12^.

Nandrolone decanoate (ND) is an anabolic androgenic steroid (AAS) used in medicine for its anabolic (tissue-building) and erythropoietic (red blood cell–stimulating) properties. It is a synthetic derivative of testosterone with lower androgenic and higher anabolic activity. ND were promotes bone mineral density in postmenopausal women and elderly men by increasing calcium retention and protein synthesis in addition to use to treat muscle wasting in patients with chronic illnesses such as HIV/AIDS and cancer^6,13^. However, chronic and unregulated use can lead to hepatic toxicity, thyroid function alteration, and cardiovascular toxicities^14–17^.

Nanoparticles, small, 1–10 nanometers in size, are highly valuable due to their unique properties, high surface area-to-volume ratio, and molecular interaction, making them valuable in biomedical, pharmaceutical, and environmental fields. Curcumin (Cur), a natural polyphenolic compound extracted from the rhizomes of *Curcuma longa* (turmeric), has been used for over 4000 years in traditional medicine due to its well-documented antioxidant, anti-inflammatory, and anticarcinogenic properties^18,19^. Extensive research has demonstrated its therapeutic potential in a variety of disorders, including arthritis, hepatic diseases, neurodegenerative disorders, obesity, and various types of cancer^20,21^.

However, despite its promising pharmacological profile, the clinical application of curcumin is significantly limited by its low bioavailability, which results from poor aqueous solubility, limited gastrointestinal absorption, rapid metabolism, and poor penetration across the blood–brain barrier. Consequently, these pharmacokinetic limitations have hindered its efficacy in vivo.

To overcome these challenges, various curcumin delivery systems have been developed to enhance its bioavailability, stability, tissue distribution, and circulation half-life. These formulations include chemical derivatives, structural analogs, and advanced nanocarrier-based systems such as nanoparticles (NPs), liposomes, micelles, nanostructured lipid carriers, and phospholipid complexes, all of which aim to improve its therapeutic efficacy^22–24^.

Chitosan, a pH-sensitive, polymeric drug delivery carrier, is widely utilized in pharmaceutical formulations due to its high safety profile, positive charge density, wound-healing capabilities, and excellent mucoadhesive properties^25,26^. It is also biocompatible, biodegradable, and exhibits low immunogenicity, making it a favorable material for biomedical applications. A variety of carrier systems have been developed to enhance the delivery and therapeutic efficacy of curcumin, including solid lipid nanoparticles, polymeric nanoparticles, biodegradable microspheres, phospholipid complexes, liposomes, and other macromolecular platforms^27^.

To the best of our knowledge, this is the first study to investigate the hepatoprotective effects of a novel nano-formulation—nano ferrite–chitosan–curcumin nanoparticles (NF-CH-CurNPs)—against nandrolone decanoate-induced liver toxicity in rats. This approach aims to combine the antioxidant and anti-inflammatory properties of curcumin with the magnetic properties of ferrite and the controlled release capabilities of chitosan to enhance therapeutic outcomes.

The aim of this study was to evaluate the therapeutic potential of nano ferrite chitosan curcumin nanoparticles (NF-CH-CurNPs) in mitigating nandrolone decanoate (ND) induced hepatic toxicity, focusing on liver function, oxidative stress, inflammation, DNA damage, and cellular proliferation in male rats.

## Materials and methods

### Chemicals and kits

All chemicals were purchased from sigma and Aldrich co.

### Nandrolone decanoate (ND)

ND is found under the name of nandurabolin (50 mg/ml 1 ampoule of 1 ml) was perchased from El Nile company, Egypt (CAS No.: 360-70-3) and it has been prepared by dissolving in olive oil.

### Curcumin nanoparticles (CurNPs)

CurNPs (Product ID: XYZ123; particle size < 100 nm) were obtained from Nawah Scientific (Cairo, Egypt).

### Chitosan

Chitosan was obtained from Alpha Chemica, Mumbai, India (Chitosan CAS No.: 9012-76-4).

### Preparation of X-type hexagonal ferrite nanoparticles

Sr_2_NiCoAl_0.3_Fe_27.7_O_46_ X-Type hexaferrite nanoparticles were prepared by the chemical Co-precipitation method^28^.

The nanocrystalline Sr_2_NiCoAl_0.3_Fe_27.7_O_46_ X-Type Hexagonal ferrite nanoparticles were prepared by the chemical Co-precipitation method according to the following equations:

$$\begin{gathered} 2{\text{SrCl}}_{2} \cdot 6{\text{H}}_{2} {\text{O}} + {\text{NiCl}}_{2} \cdot 6{\text{H}}_{2} {\text{O}} + {\text{CoCl}}_{2} + 0.3{\text{Al}}\left( {{\text{NO}}_{3} } \right)_{3} \cdot 9{\text{H}}_{2} {\text{O}} + 27.7{\text{ FeCl}}_{3} \hfill \\ \quad + 92\;{\text{NaOH~Sr}}_{2} {\text{NiCoAl}}_{{0.3}} {\text{Fe}}_{{27.7}} {\text{O}}_{{46}} + 91.1{\text{NaCl}} + 0.9{\text{NaNO}}_{3} + 66.7{\text{H}}_{2} {\text{O}} \hfill \\ \end{gathered}$$

Stoichiometric amounts of Metal chlorides and nitrates are dissolved in distilled water and kept at 10 °C for 1 h. The reactants are constantly stirred using a magnetic stirrer. The PH of the mixed solution was constantly monitored as NaOH solution was added drop wise until PH reached 12. Then the solution was heated and maintained at 80 ° C for 2 h under continuous stirring. The precipitates are thoroughly washed with distilled water until the washings become free from sodium chloride and sodium nitrate. The precipitates are dried for a few days at room temperature. Then the samples are thoroughly ground in an agate mortar to obtain ultra-fine powder. Thereafter, the samples were heated at 1150 ° C for 5 h and then left to gradually cooled to room temperature, then ground in an agate mortar to obtain ultra-fine powder.

### Functionalization of X-type hexagonal with Chitosan (CH)

A 0.5% (w/v) chitosan solution was prepared by dissolving chitosan in (2% V/V) acetic acid at PH 5 under continuous stirring. 100 mg of Sr₂NiCoAl₀.₃Fe₂₇.₇O₄₆ X-type hexagonal ferrite nanoparticles were then added to the chitosan solution. The resulting mixture was subjected to sonication at 60 °C for 1 h to ensure uniform dispersion, followed by mechanical stirring (~ 1000 rpm) at room temperature for 18 h to promote interaction between chitosan and ferrite nanoparticles. The ferrite-chitosan nanocomposite was then separated using an external magnetic field and dried overnight, following the method described by Ramnandan et al.^29^

### Construction of NF-CH-CurNPs

Curcumin (50.0 mg) was dissolved in 10 mL of anhydrous ethanol, and the early prepared Sr_2_NiCoAl_0.3_Fe_27.7_O_46_ X-Type Hexagonal ferrite nanoparticles/CH nanocomposites were added to this solution, with the ratio between Sr_2_NiCoAl_0.3_Fe_27.7_O_46_ X-Type Hexagonal ferrite and Curcumin is 2:1. The mixture was stirred at 1000 rpm at room temperature for 18 h. Subsequently, the resulting product was alternating washed with anhydrous ethanol and distilled water, followed by magnetic separation to eliminate any remaining raw materials. Finally, the precipitate was dried, and the magnetic Sr_2_NiCoAl_0.3_Fe_27.7_O_46_ X-Type Hexagonal ferrite nanoparticles/CH/Curcumin i.e. (NF-CH-Curcumin) nano-system was obtained.

### Vibrating sample magnetometer (VSM) measurements

Hysteresis loops of the samples were recorded at room temperature using the vibrating sample magnetometer, Lakeshore – 7410 and a maximum applied field up to 20,000 Gauss.

### Characterization of nanoparticles

The surface morphology and size of nanoparticles was determined using Scanning Electron Microscopy (SEM) and Transmission Electron Microscopy (TEM) and other images confirm our results (Data found in Supplementary File). Scanning Electron Microscopy (SEM) with Acceleration Voltage 30 KV (SEM; JeoL-JSM- 6510, Japan). SEM was carried out using sputtering technique, specimens were coated by an Au thin film then observation was carried out. High resolution Transmission Electron Microscopy (TEM) with Acceleration Voltage 200 KV (HTEM; JeoL-JEM- 2100 PLUS, Japan), where the sample powder was first dispersed in pure ethanol by ultrasonic waves for 60 min to perfectly separate the nanoparticles from each other and then the suspension was dropped on a copper grid with a carbon film.

The crystalline nature of the prepared nanoparticles was confirmed by X-ray diffraction analysis (XRD) with scanning range 2θ = 20–80, step size = 0.02 using GNR APD 2000 Pro X-ray diffractometer step scan type and CuK_α1_ radiation with wavelength λ = 1.540598 Å. In addition, FTIR spectra were recorded at room temperature using Bruker Tensor 27 FT-IR Spectrometer in the range 200 to 4200 cm^−1^ to verify the NF-CH-CurNPs synthesis. Moreover, Hysteresis loops of the samples were recorded at room temperature using the vibrating sample magnetometer, Lakeshore – 7410 and a maximum applied field up to 20,000 Gauss.

### Experimental animals and ethics declarations

All animal procedures were performed in accordance with ethical standards for the use of laboratory animals in research, as approved by the Institutional Animal Care and Use Committee (IACUC) at the Faculty of Science, Tanta University, under license number IACUC-SCI-TU-0381. The study also complied with the guidelines outlined by the U.S. National Institutes of Health (NIH) for the care and use of laboratory animals. ^30^ All methods were reported following the ARRIVE guidelines, and all data and materials are available upon request. A total of 48 adult male albino rats (*Rattus norvegicus*), weighing 175 ± 15 g, were obtained from the National Research Center, Egypt. The animals were housed under standard laboratory conditions (temperature 22 ± 2 °C, 12-h light/dark cycle) with free access to a commercial diet and water.

### Experimental design

Rats were randomly divided into 6 groups.

- Group 1 acts as the control were healthy mice were administrated with vehicle (sterile physiological saline) for 2 weeks.
- Group 2 was CurNPs were rats received 50 mg/kg body weight/2 day CurNPs with oral gavage for two weeks^28,30^.
- Group 3 was NF-CH-CurNPs in which rats received 24 mg/Kg body weight/2 day NF-CH-CurNPs for 2 weeks with oral gavage.
- Group 4 was ND in which rats received ND (25 mg/Kg body weight/week) with oral gavage for four weeks. ^13^
- Group 5 was ND + CurNPs in which rats received ND for 4 weeks then treated with CurNPs for another 2 weeks.
- Group 6 was ND + NF-CH-CurNPs in which rats received ND for 4 weeks then treated with NF-CH-CurNPs for another 2 weeks.

#### Euthanasia

At the end of the experiment, rats were euthanized by an overdose of sodium pentobarbital (150 mg/kg, intraperitoneally), consistent with the AVMA Guidelines for the Euthanasia of Animals.

#### Blood and serum samples

Rats were then slaughtered after being given sodium pentobarbital anesthesia at the conclusion of the research. Blood samples were drawn aseptically via a venipuncture and placed in a dry, clean, and sterile tube without the use of any anticoagulants, allowing the blood to clot. Blood samples were centrifuged for 5 min at 4000 rpm after being allowed to stand for 20 min at 4 °C to allow for coagulation. The obtained serum was stored at − 18 °C until a blood parameter was determined. Post decapitation, rats were dissected, and the liver were promptly extracted and halved. Small liver sample was preserved in a 10% neutral buffered formalin solution for subsequent histopathological and immuohistochemical examination. The remaining portion of the liver sample was weighed and homogenized to facilitate various estimations.

### Biochemical assays

Using a commercial kit provided by Randox (Egypt), the activity of alanine transaminase (ALT) and aspartate transaminase (AST) were estimated after Reitman and Frankel^31^. Belfield and Goldberg^32^ used a commercial kit from France’s BioMérieux Co to measure the activity of alkaline phosphatase (ALP). Using commercial diagnostic kits provided by Diamond (Egypt) and spectrophotometric analysis, the quantities of albumin and total proteins in serum were measured after Bowers and Wong^33^ and Doumas et al. ^34^, respectively.

### Assessment of oxidative stress indicators

The study evaluated oxidative stress levels in liver homogenates using biodiagnostic analyze kits, focusing on lipid peroxidation (malondialdehyde; MDA) activity described by Wided et al.^35^ method.

### Evaluation of the antioxidant enzymes

The antioxidant activities were assessed through the evaluation of some enzymes such as.

reduced glutathione activities described by Mesbah et al.^36^ method while catalase enzyme (CAT), and superoxide dismutase enzyme activity (SOD) by means of the commercial kits and according to the technique defined by Saggu et al.^37^ and Misra et al.^38^ respectively.

### Histopathological examination

Liver tissues were collected from all experimental rats at the end of the study. From each animal, one liver lobe was fixed in 10% neutral buffered formalin for at least 48 h, then processed using standard paraffin-embedding techniques. Multiple Sects. (4–5 μm thick) were cut from each paraffin block and stained with hematoxylin and eosin (H&E) following Tousson^39^.

### Immuno-histochemical analysis

Immuno-labeling involved loading 5 μm slices of liver onto positively charged slides. Different monoclonal anti-mouse primary antibodies were used to detect proliferating nuclear antigen (PCNA) and tumor necrosis factor alpha (TNFα) expressions in liver sections was performed according to Tousson et al.^40^ and El-Masry et al.^41^ respectively. Distribution of PCNA stained nuclei and TNFα stained cytoplasm were examined in deparaffinized sections using an Avidin–Biotin–Peroxidase immunohistochemical method (Elite–ABC, Vector Laboratories, CA, USA) with PCNA monoclonal antibody (dilution 1:100; DAKO Japan Co, Tokyo, Japan) and TNFα monoclonal antibody (dilution 1:100; DAKO Japan Co, Tokyo, Japan). The brown hue of the stained cells was recovered using Image J’s colour thresholding feature for quantitation.

### Statistical analysis

The analysis was done using the Statistical Package for the Social Sciences (SPSS software version 17). Data were expressed as the significance of difference was analysed by one-way ANOVA. Values are expressed as means ± SE. (*) and (^#^) significant difference from control ND and from groups respectively at *p*< 0.01.

## Results

### Characterization of NF-CH-CurNPs

**Fig. 1:**
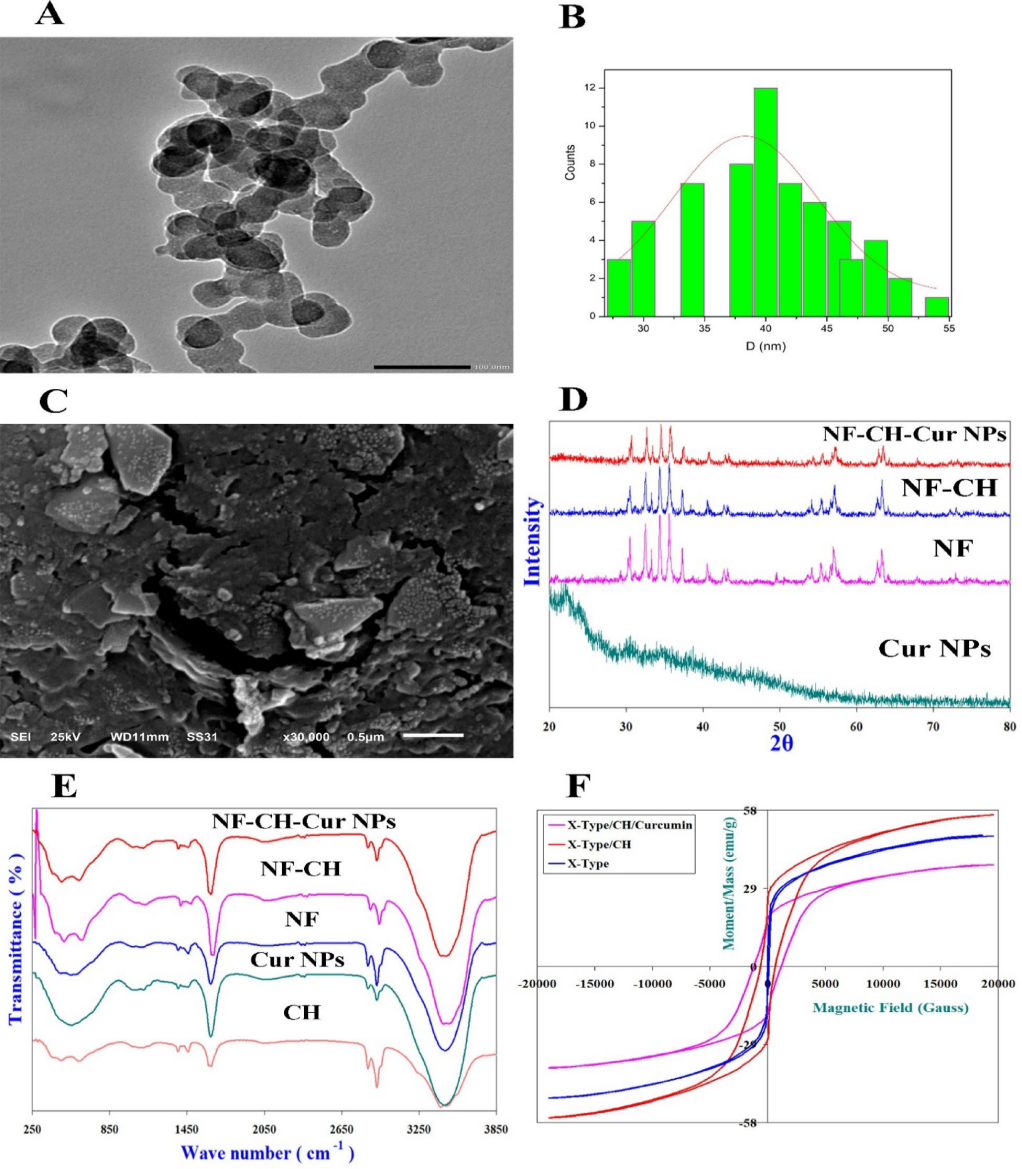
(**A**) HTEM image for NF-CH-CurNPs. (**B**) Nanoparticle size distribution. (**C**) SEM images for NF-CH-CurNPs. (**D**) XRD patterns for Cur NPs, NF, NF-CH, NF-CH-CurNPs. (**E**) FT-IR Spectra for Cur NPs, CH, NF, NF-CH, NF-CH-CurNPs. (**F**) Magnetic hysteresis loops for Sr_2_NiCoAl_0.3_Fe_27.7_O_46_ X-Type Hexagonal ferrite nanoparticles, Ferrite/CH and Ferrite/CH/CurNPs.

NF-CH-CurNPs were examined using HTEM and SEM images to determine their nanoparticle size, surface characteristics, and morphological characteristics in Fig. 1A–C. NF-CH-CurNPs had a smooth surface, were roughly spherical in shape, and their average nanoparticle size obtained from HTEM was ~ 40 nm, whereas their average grain size obtained from SEM images was ~ 33 nm (Fig. 1A–C).

### X-ray diffraction spectra

XRD patterns for Sr_2_NiCoAl_0.3_Fe_27.7_O_46_ X-Type Hexaferrites proved that they have single-phase X-Type Hexagonal nanoferrites (Fig. 1D). XRD patterns illustrated the amorphous nature of CurNPs. Furthermore, XRD patterns of Sr_2_NiCoAl_0.3_Fe_27.7_O_46_ coated with CH (NF-CH) showed some broadening in the diffraction peaks as a result to the amorphous nature of Chitosan and indicating that X-Type nanostructures is coated by CH matrix. Figure 1D reveals that CH and CurNPs does not result in phase change of Sr_2_NiCoAl_0.3_Fe_27.7_O_46_ nanostructures. The use of a CH-based cross-linked network with Sr_2_NiCoAl_0.3_Fe_27.7_O_46_ and the formation of NF-CH-CurNPs induced a widening of the emerged peaks (Fig. 1D).

### Infrared spectra

IR absorption spectra for nano-crystalline Sr_2_NiCoAl_0.3_Fe_27.7_O_46_ X-Type Hexaferrite have eight absorption bands; ν_1_, ν_2_, ν_3_, ν_4_, ν_A_, ν_B_, ν_T1_ and ν_T2_ (Fig. 1E). For X-Type Hexaferrite ν_1_, ν_2_ and ν_3_ observed at 605.63 cm^−1^, 565.127 cm^−1^ and 478.33 cm^−1^, respectively; assigned to stretching vibrations of A-site metal ion-oxygen bonding, vibrations of B-site metal ion-oxygen complexes and to the divalent ion bonds Ni^2+^–O^2−^, Co^2+^–O^2−^ and Fe^2+^–O^2−^ existed among B-sites, respectively. ν_4_ at 297.03 cm^−1^ assigned to lattice vibrations of the system^8–10^. ν_T1_ at 1637.52 cm^−1^ assigned to retained water (humidity) in the samples. ν_A_ and ν_B_ at 887.23 and 1053.11 cm^−1^, respectively. ν_A_ assigned to increase in concentration of Fe^2+^, Sr^2+^ and/or Co^2+^ among A-sites. ν_B_ assigned to Fe^4+^–O^2−^ bonds. ν_T2_ at ~ 3465.99 cm^−1^ imputed to H-O-H stretching vibrational confirming adsorption of water in the samples. Characteristic CH peaks appears around 1487.07 and 1660.66 cm^−1^ of amino and amide groups. For CH, its bands at 3504.65 cm^−1^, 1660.66 cm^−1^, 1407.99 cm^−1^ and 1083.97 cm^−1^ related to axial stretching of O-H group, which appears superimposed to N-H stretching, C = O stretching of acetyl units, N-H group and C-O and C-O-C stretching, respectively. For CurNPs, its bands observed at 1128.33 cm^−1^ (C–O–C stretching), 1641.38 cm^−1^ (C–O Stretching) and 3477.56 cm^−1^ (OH stretching). IR spectra of NF-CH-CurNPs (Fig. 1E) demonstrated the characteristic bands of CH and CurNPs confirming the existence of CH and CurNPs in the Nano-system.

### Magnetic hysteresis loops

Room temperature hysteresis loops for the nano-crystalline Sr_2_NiCoAl_0.3_Fe_27.7_O_46_ X-Type Hexagonal ferrite nanoparticles (Fig. 1F) indicated a narrow hysteretic behavior (low coercivity Hc) which is characteristic of ferrimagnetic materials, with high saturation magnetization Ms = 48.835 emu/g, low coercivity Hc is 50.102 Gauss, low remanent magnetization Mr = 6.37 emu/g and small squareness Mr/Ms = 0.13044.

The uncoated Sr_2_NiCoAl_0.3_Fe_27.7_O_46_ exhibited a high saturation magnetization (MS), which increased with Chitosan (CH) coating to 56.23 emu/g. Sr_2_NiCoAl_0.3_Fe_27.7_O_46_/CH revealed a significant shielding effect by CH. Hc were observed to increase upon coating with CH reaching 585.04 Gauss. Materials with high magnetic anisotropy usually exhibit high coactivity i.e. they are hard to demagnetize. Hc increased upon CH coating, conferring a hardening or binding effect on Sr_2_NiCoAl_0.3_Fe_27.7_O_46_. Conversely, a high change was noticed in magnetic Properties (Ms = 37.709 emu/g and Hc = 1199.9 Gauss) for Sr_2_NiCoAl_0.3_Fe_27.7_O_46_/CH/Cur, which may be attributed to the reduced surface defects on Sr_2_NiCoAl_0.3_Fe_27.7_O_46_ and a dilution effect of Cur.

#### Effect of NF-CH-CurNPs on liver functions

Table (1) revealed that; a significant (*P* < 0.01) elevation in serum AST, ALT, ALP, total protein and a significant (*P* < 0.01) depletion in albumin in ND group as compared with control. However, treatment of rats by ND with CurNPs or/and NF-CH-CurNPs showed a significant (*P* < 0.01) depletion in AST, ALT, ALP, total protein and a significant (*P* < 0.01) elevation in albumin as compared to ND with best results for NF-CH-CurNPs treatment (Table 1).

### Effect of NF-CH-CurNPs on liver oxidative stress

Table (2) revealed that; a significant (*P* < 0.01) elevation in liver MDA and a significant (*P* < 0.01) depletion in GSH, CAT, SOD in ND group as compared with control. However, treatment of rats by ND with CurNPs or/and NF-CH-CurNPs showed a significant (*P* < 0.01) depletion in MDA and a significant (*P* < 0.01) elevation in GSH, CAT, SOD as compared to ND with best results for NF-CH-CurNPs treatment (Table 2).

**Table 1:** Changes in the levels of liver functions in different groups under study.

	ALT (U/L)	AST (U/L)	ALP (U/L)	T. protein (g/dl)	Albumin (mg/dl)
Control	33.40^#^ ±1.63	39.10^#^ ±2.46	97.3^#^ ±4.80	5.24^#^ ± 0.15	4.25^#^ ±0.22
CurNPs	30.85^#^ ±1.14	45.60^#^ ±2.75	104.5^#^ ±4.10	5.13^#^ ± 0.09	4.43^#^ ±0.15
NF-CH-CurNPs	31.50±^#^ 1.37	45.30^#^± 2.43	105.0^#^ ± 3.61	5.09 ^#^ ±0.08	4.67^#^± 0.07
ND	62.60*±1.66	81.95*±2.68	201.9*±4.62	6.20*±0.11	3.67* ±0.15
ND + CurNPs	52.25^#^* ±2.05	62.13^#^*±2.55	170.1^#^* ±3.59	5.85^#^* ±0.11	4.18^#^± 0.18
ND + NF-CH-CurNPs	44.58^#^* ±0.96	40.07^#^ ±1.97	122.5^#^* ±2.50	6.03* ±0.05	4.29^#^±0.11

The significance of difference was analyzed by one – way ANOVA using computer program. *N* = 8 rats; values are expressed as means ± SEM. ^#^ and * significant difference from ND and control group respectively at *P* < 0.01.

**Table 2:** Changes in the oxidative stress in liver homogenates in different groups under study.

	MDA (nmol/g tissue)	GSH (mmol/g tissue)	SOD (U/g tissue)	CAT (nmol/g tissue)
Control	35.57^#^± 0.75	13.13^#^± 0.21	79.50^#^± 0.52	29.60^#^ ±0.64
CurNPs	33.50^#^± 0.61	12.83^#^± 0.20	82.13^#^± 0.52	28.77^#^±0.18
NF-CH-CurNPs	31.60^#^±0.49	16.78^#^± 0.06	87.50^#^± 0.61	33.77^#^±0.23
ND	103.5*± 4.52	7.76*± 0.20	39.37*± 1.30	17.33*±0.12
ND + CurNPs	80.30^#^*±0.76	9.133^#^*± 0.23	53.03^#^*± 1.07	21.83^#^*±0.85
ND + NF-CH-CurNPs	42.30^#^±0.51	12.20^#^± 0.12	75.00^#^± 0.79	27.20^#^±0.40

The significance of difference was analyzed by one – way ANOVA using computer program. *N* = 8 rats; values are expressed as means ± SEM. ^#^ and * significant difference from ND and control group respectively at *P* < 0.01.

### Histopathological examination

**Fig. 2:**
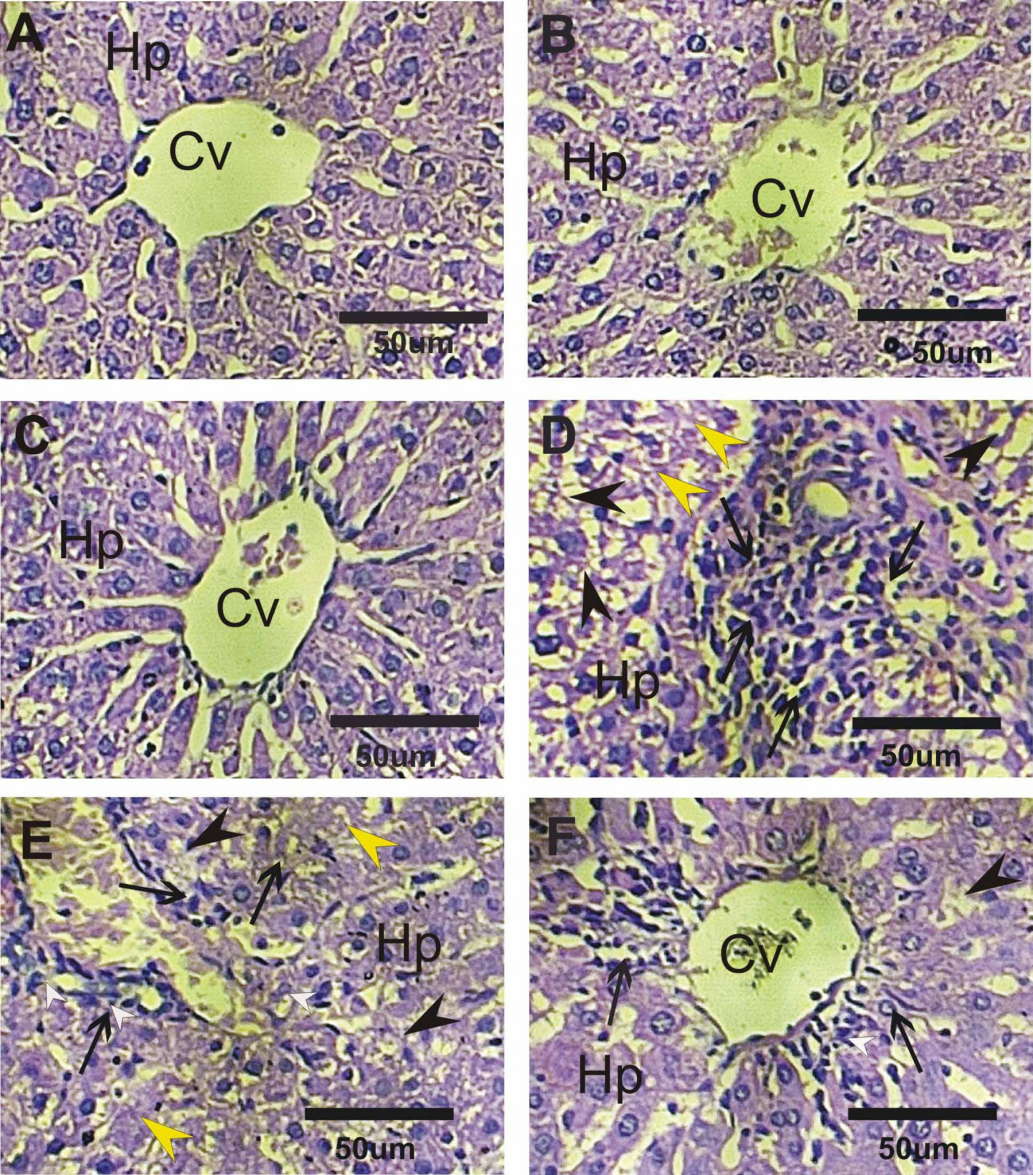
Liver sections of different groups stained with H & E. (**A**–**C**) Standard structure of hepatocytes (Hp) and normal central veins (CV) in control, CurNPs and NF-CH-CurNPs groups. (**D**) Marked cytoplasmic vacuolization of hepatocytes (black arrow heads) with marked inflammatory cells (arrows), and marked degeneration with focal area and central zonal necrosis (yellow arrow heads) in liver sections in ND group. (**E**) Liver sections in ND + CurNPs revealed moderate hepatocellular vacuolation, moderate to mild inflammatory cell infiltrations (arrows), mild necrosis, and marked degeneration. (**F**) Mild cytoplasmic vacuolization of hepatocytes and mild inflammatory cell infiltrations in liver in ND + NF-CH-CurNPs.

Haematoxylin and eosin stained livers sections in treated rats with CurNPs, NF-CH-CurNPs or control showed radially arranged cords of polygonal hepatocytes that extend from a central vein to the periphery of the hepatic lobules at which the portal tracts appears (Fig. 2A–C). Liver sections in treated rats with ND showed disturbance of the hepatocytes radially arranged cords, marked hepatocellular vacuolation, marked inflammatory cell infiltrations, moderate fibrosis, marked degeneration with focal area and central zonal necrosis were evident (Fig. 2D). On the other hand liver sections in treated rats with ND + CurNPs or ND + NF-CH-CurNPs revealed enhancment in liver structure as compared to ND with best results for NF-CH-CurNPs treatment (Fig. 2E and F). Liver sections in treated rats with ND + CurNPs revealed moderate hepatocellular vacuolation, moderate to mild inflammatory cell infiltrations, mild fibrosis, and marked degeneration while liver sections in treated rats with ND + NF-CH-CurNPs revealed mild inflammatory cell infiltrations and mild hepatocellular vacuolation (Fig. 2E and F).

### Immuno-histochemical localization

**Fig. 3:**
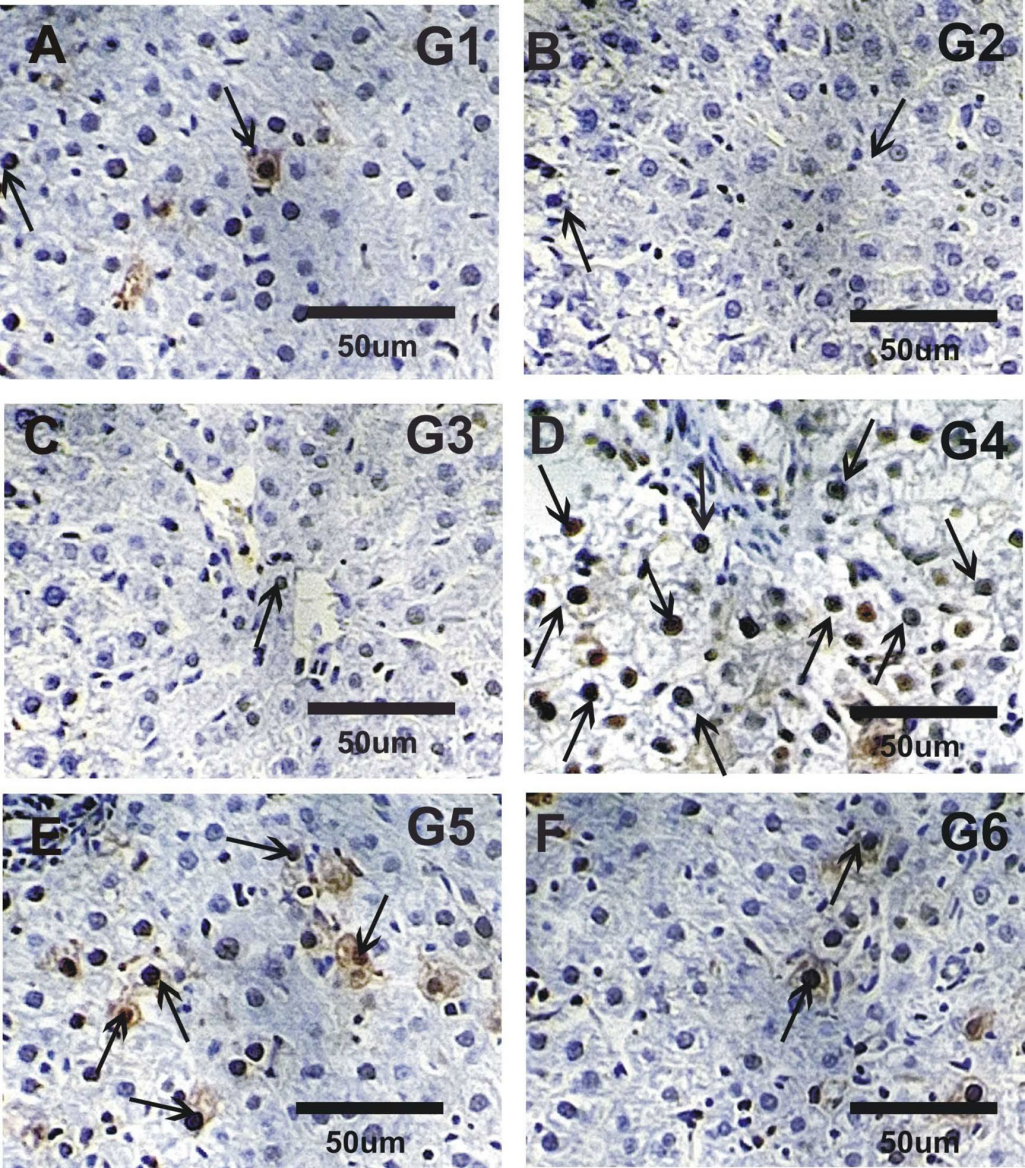
Liver sections photo micrographs stained with PCNA in studies groups. (**A**–**C**) Faint positive reaction for PCNA expressions in control (G1), CurNPs (G2) and NF-CH-CurNPs (G3). (**D**) Strong reaction (arrows) for PCNA in ND (G4). (**E**, **F**) Liver sections in G5 (ND + CurNPs) and G6 (ND + NF-CH-CurNPs) moderate to mild PCNA reactions respectively.

Figures 3 and 5 revealed the immuno-histochemical localization of PCNA in liver tissues. Liver sections in control, CurNPs, NF-CH-CurNPs groups revealed faint to mild reaction while strong reaction was detected in ND (Fig. 3A–D). Liver sections in treated rats with ND + CurNPs and ND + NF-CH-CurNPs revealed moderate to mild PCNA reactions respectively (Fig. 3E, F).

**Fig. 4:**
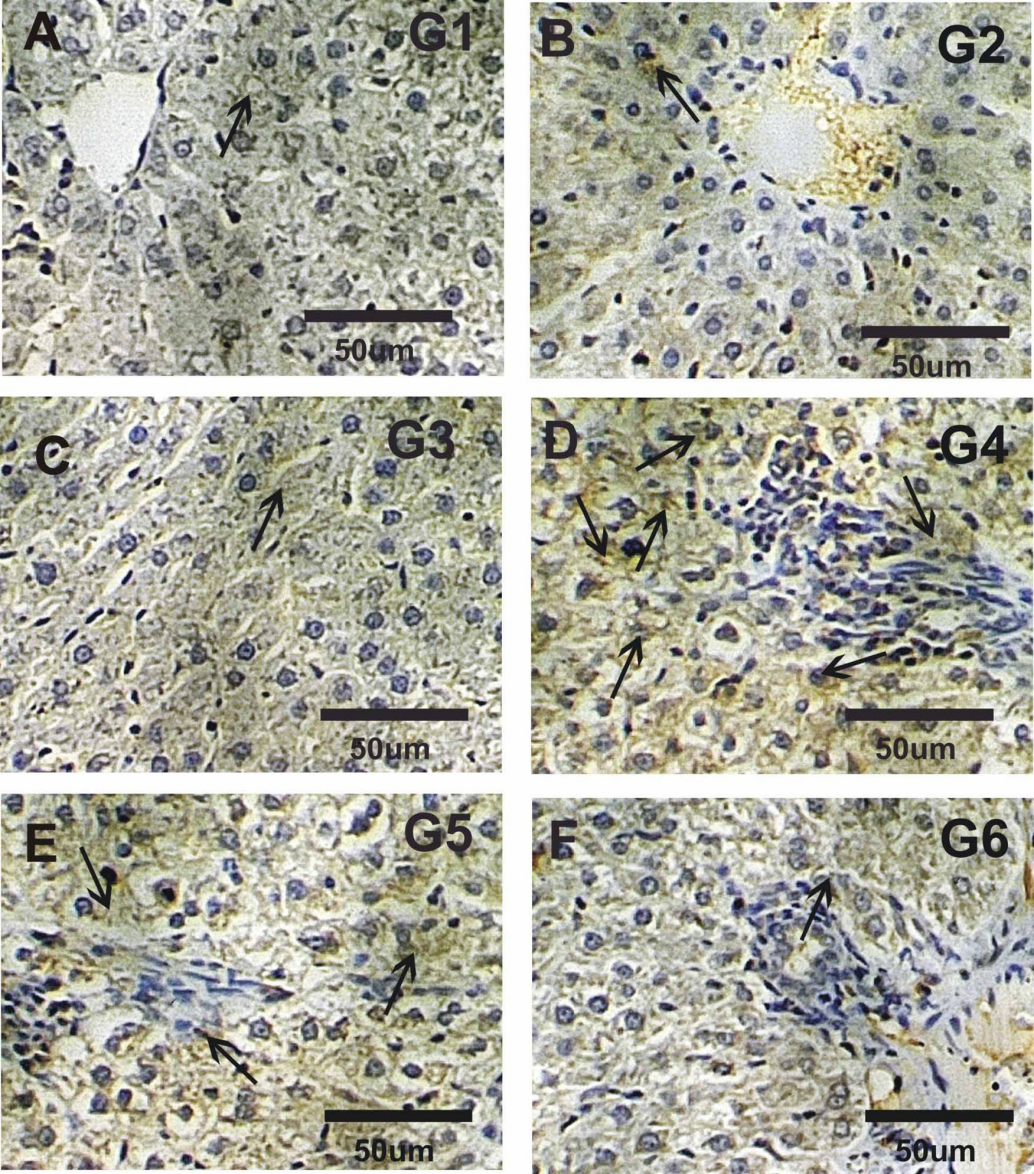
Liver sections photo micrographs stained with TNFα in studies groups. (**A**–**C**) Negative reaction for TNFα in control (G1), Cur NPs (G2) and NF-CH-CurNPs (G3) groups. (**D**) Strong reaction (arrows) for TNFα in ND (G4). (**E**, **F**) moderate to mild positive TNFα reactions in G5 (ND + CurNPs) and G6 (ND + NF-CH-CurNPs) respectively.

**Fig. 5:**
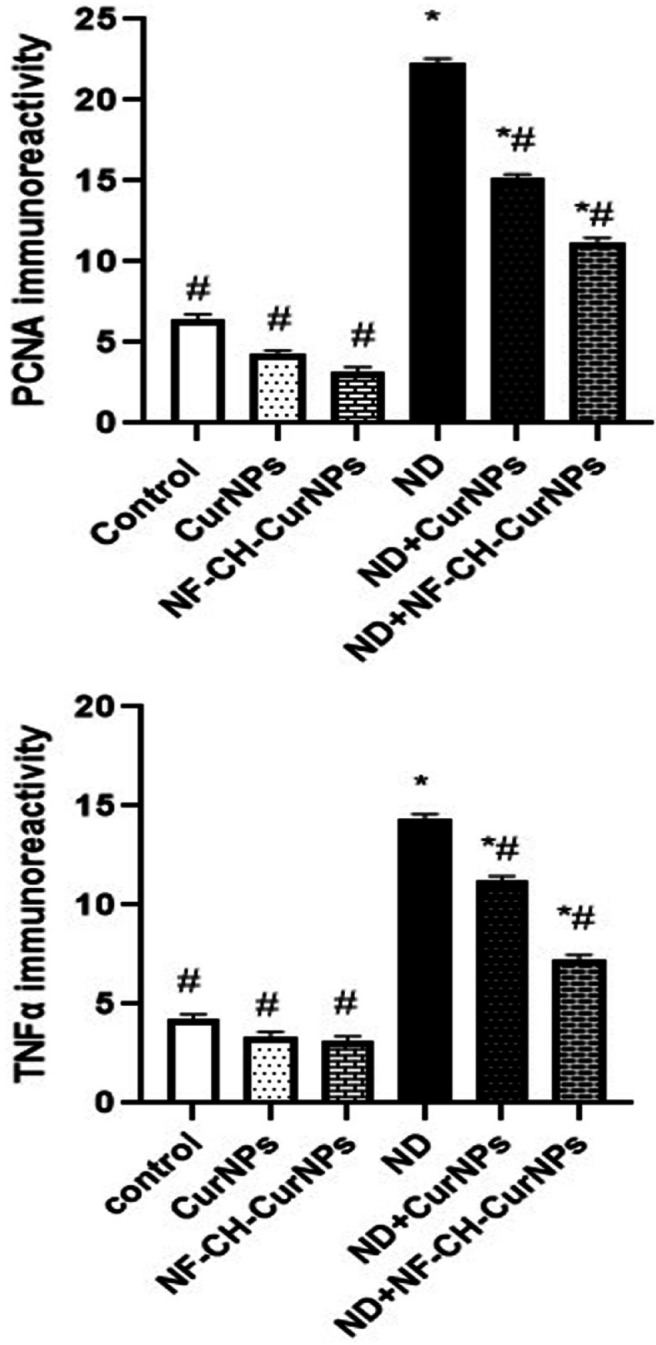
Changes in PCNA and TNFα expressions in liver sections in the studies groups. The significance of difference was analyzed by one-way ANOVA using computer program. Values are expressed as means ± SEM. ^#^ and *significant difference from ND and control groups respectively at *P* < 0.01.

Figures 4 and 5 revealed the immuno-histochemical localization of TNFα in liver tissues. Liver sections in control, CurNPs, NF-CH-CurNPs groups revealed faint reaction while strong reaction was detected in ND (Fig. 4A–D). Liver sections in treated rats with ND + CurNPs and ND + NF-CH-CurNPs revealed moderate to mild PCNA reactions respectively (Fig. 4F).

## Discussion

Injectable AASs can be used by athletes to directly build and gain muscle mass, women also use it in a variety of cosmetics to enhance the appearance of their breasts, buttocks, cheekbones, and lips^42,43^. Additionally, humans may be indirectly impacted by consuming meat from animals that have had AAS therapy. Nandrolone Decanoate, one of the AAS that their effects on cellular levels and hepatic tissue are still being studied and are not entirely understood, Therefore, the current study aimed to examine the protective effects of nano ferrite chitosan curcumin nanoparticles (NF-CH-CurNPs) against liver toxicity and oxidative stress induced by the novel steroid nandrolone decanoate (ND) in rats. Current results revealed that; ND induced liver toxicity in rats and the treatments of ND with NF-CH-CurNPs (ND + NF-CH-CurNPs) or/and with CurNPs (ND + CurNPs) improved these changes in liver functions with best results for the treatments with NF-CH-CurNPs; these results agree with Tousson et al.^2^ who find that, growth promoter induced liver toxicity by increasing ALT, AST and decreasing albumin levels in rabbits. Similarly, current results agree with Bond et al.^44^; El-Halwagy et al.^46^ who reported that; anabolic androgenic steroid-induced hepatotoxicity. Current results are in agreement with El-Gizawy et al.^46^ who reported that; CurNPs ameliorate hepatotoxicity and nephrotoxicity induced by cisplatin in rats. Also current results are in agreement with Alghriany et al.^47^ who reported that; CurNPs ameliorate aluminum oxide nanoparticles induced liver toxicity.

Oxidative stress arises from an imbalance between pro-oxidants and antioxidants, leading to cellular damage due to the depletion of endogenous antioxidants, insufficient dietary intake, and excessive production of free radicals^2,23^. The toxicities caused by AASs have been directly linked to oxidative stress, which results in lipid peroxidation, glutathione depletion, and other oxidant processes that damage and kill biological macromolecules, particularly the cell membrane^48^. The current investigation showed that, in comparison to the control group, ND dramatically reduced the means of GSH, CAT, and SOD and greatly raised the mean value of MDA. Conversely, co-treatment with CurNPs (ND + CurNPs) or nano ferrite-chitosan-curcumin nanoparticles (ND + NF-CH-CurNPs) markedly attenuated these oxidative stress markers, with NF-CH-CurNPs demonstrating the most pronounced protective effect. Current results have been shown that; NF-CH-CurNPs have the capacity to scavenge free radicals. Current results are in agreement with Ranjbar et al.^49^ who find that; CurNPs and Cur improved the oxidant and antioxidant system in the liver mitochondria after aluminum phosphide.

These findings are consistent with those of Frankenfeld et al.^50^, who proposed that nandrolone induces oxidative stress in the kidney, liver, and heart of male rats. Similarly, Dornelles et al.^51^ reported oxidative stress in the liver and kidneys following anabolic steroid administration. Moreover, Hussain et al.^4^ observed that Boldenone triggered myocardial oxidative stress in male rats, further supporting the current study’s findings.

CurNPs therapy may have restored hepatic GSH levels because Cur has been shown to stimulate GSH production by upregulating glutamylcysteine ligase expression, which in turn raises glutamylcysteine ligase activity^52^. Current results align with the research conducted by Ahmed et al.^53^, which revealed that CurNPs regulate oxidative damage in rat liver. Current findings agree with those of Shelash et al.^54^, who show that CurNPs cause ROS-mediated apoptosis and prevent human liver cancer cells from migrating. Current results are in agreement with Yadav et al.^55^ who find that; CurNPs prevents oxidative stress induced by arsenic and fluoride in rats.

Liver sections from the ND-treated group showed disrupted radial arrangement of hepatocyte cords and significant cellular damage, including pronounced hepatocellular vacuolation, extensive inflammatory cell infiltration, marked necrosis, severe degeneration, and elevated expression of PCNA and TNFα. Treatments of ND with NF-CH-CurNPs (ND + NF-CH-CurNPs) or/and with CurNPs (ND + CurNPs) improved these histopathological changes and deplete the elevation in PCNA and TNFα expressions with best results for the treatments with NF-CH-CurNPs. Therefore, NF-CH-CurNPs is an immunomodulatory and anti-inflammatory drug that prevents the generation of associated cytokines as well as the growth and differentiation of inflammatory cells. These results are also consistent with earlier research by Tousson et al.^40^ who reported that boldenone induced liver cells injury. This was supported by a study by Al-Aubody and Al-Diwan^56^ that revealed a higher incidence of liver abnormalities, such as hepatocyte necrosis and central vein congestion, in rats given AAS for 12 weeks. Similarly, Yahya et al.^57^ demonstrated that thioacetamide (TAA) toxicity significantly upregulated TNFα gene expression in liver tissue. Current study’s results are consistent with those of Radwan et al.^59^, who found that; curcumin-loaded nanoparticles protect against thioacetamide-induced acute liver injury and decrease PCNA immunoreactivity. Furthermore, Zhou et al.^59^, confirmed the anti-inflammatory potential of CurNPs, noting their suppressive effect on TNF-α through downregulation of its gene expression.

## Conclusion

The findings of this study demonstrate that ND administration induces significant hepatic toxicity, marked by elevated liver enzymes, oxidative stress markers, inflammatory cytokines, DNA damage, and cell proliferation. Treatment with CurNPs or NF-CH-CurNPs significantly ameliorated these adverse effects, with NF-CH-CurNPs showing superior protective efficacy, likely due to their enhanced antioxidant and anti-inflammatory properties. The novelty lies in the synthesis of a multifunctional nanocomposite that enhances curcumin’s bioactivity, providing superior antioxidant, anti-inflammatory, and DNA-protective effects compared to curcumin nanoparticles alone. These results suggest that NF-CH-CurNPs may serve as a promising therapeutic agent against ND-induced liver damage.

## Supplementary Information

Below is the link to the electronic supplementary material.

Supplementary Material 1

## Data Availability

The data presented in this study are available on request from the corresponding author.
