# Supplementary material for: Ferrite chitosan curcumin nanoparticles alleviate nandrolone decanote induced liver toxicity in male albino rats
Source: Sci Rep. 2025 Oct 29;15:37740. doi: 10.1038/s41598-025-21814-9 (PMC12572379; doi:10.1038/s41598-025-21814-9)
Supplement: Supplementary file 1 — Supplementary Material 1 [file 41598_2025_21814_MOESM1_ESM.docx]

First; Regarding SEM and TEM measurements: we have measured SEM and High Resolution TEM (HTEM) for the second time in other universities in Egypt using another apparatus as follows:

1- The surface morphology and size of nanoparticles was determined using **Scanning Electron Microscopy with Acceleration Voltage 30 KV (SEM;** JeoL-JSM- 6510, Japan**).** SEM was carried out using sputtering technique, specimens were coated by an Au thin film then observation was carried out.

2- High Resolution T**ransmission Electron Microscopy with Acceleration Voltage 200 KV (HTEM;** JeoL-JEM- 2100 PLUS, Japan), where the sample powder was first dispersed in pure ethanol by ultrasonic waves for 60 min to perfectly separate the nanoparticles from each other and then the suspension was dropped on a copper grid with a carbon film.

Second; we have accurately measured the grain size for NF-CH-CurNPs from SEM and its average value was ~ 33 nm. And we have measured the nanoparticle size for NF-CH-CurNPs and its average value was ~ 40 nm. This is expected as nanoparticle size is generally greater than grain size. Furtheremore we have plotted the nanoparticle size distribution obtained from HTEM image for NF-CH-CurNPs and this diagram confirmed the obtained results.

Third; for DLS ; I want to inform you that this measure is not available at the current time.

The following images confirm our results:


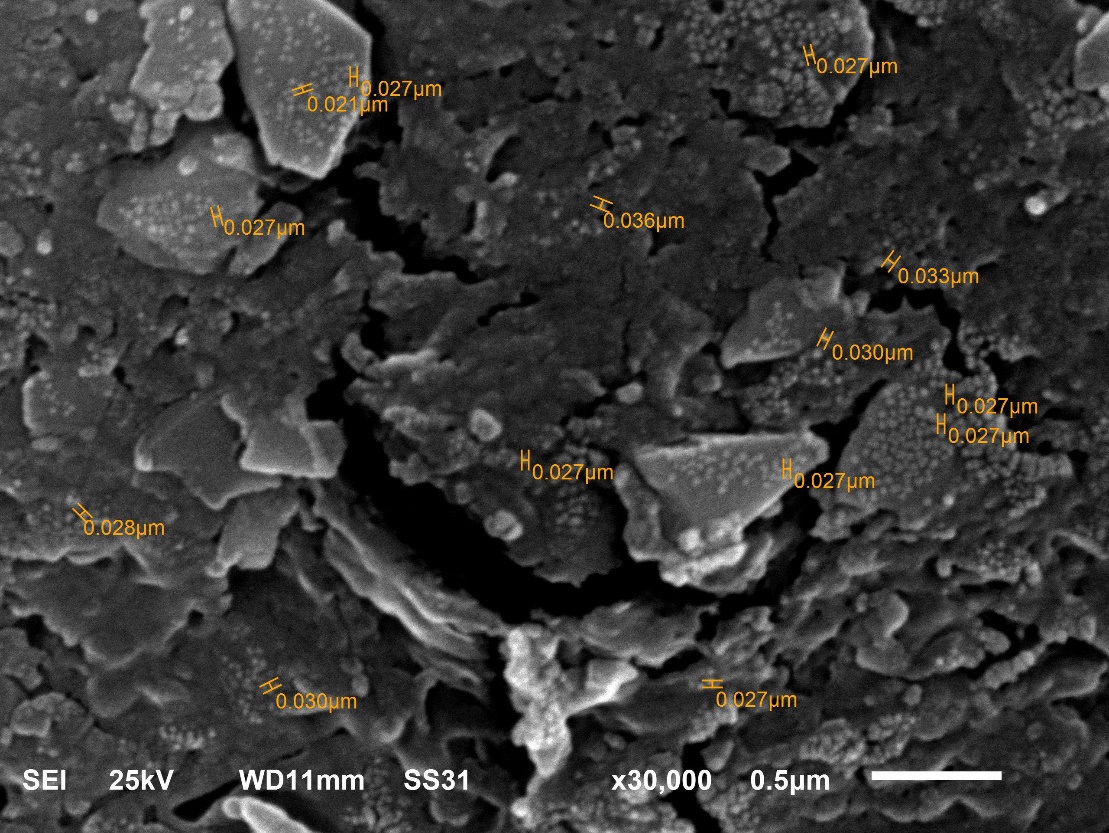


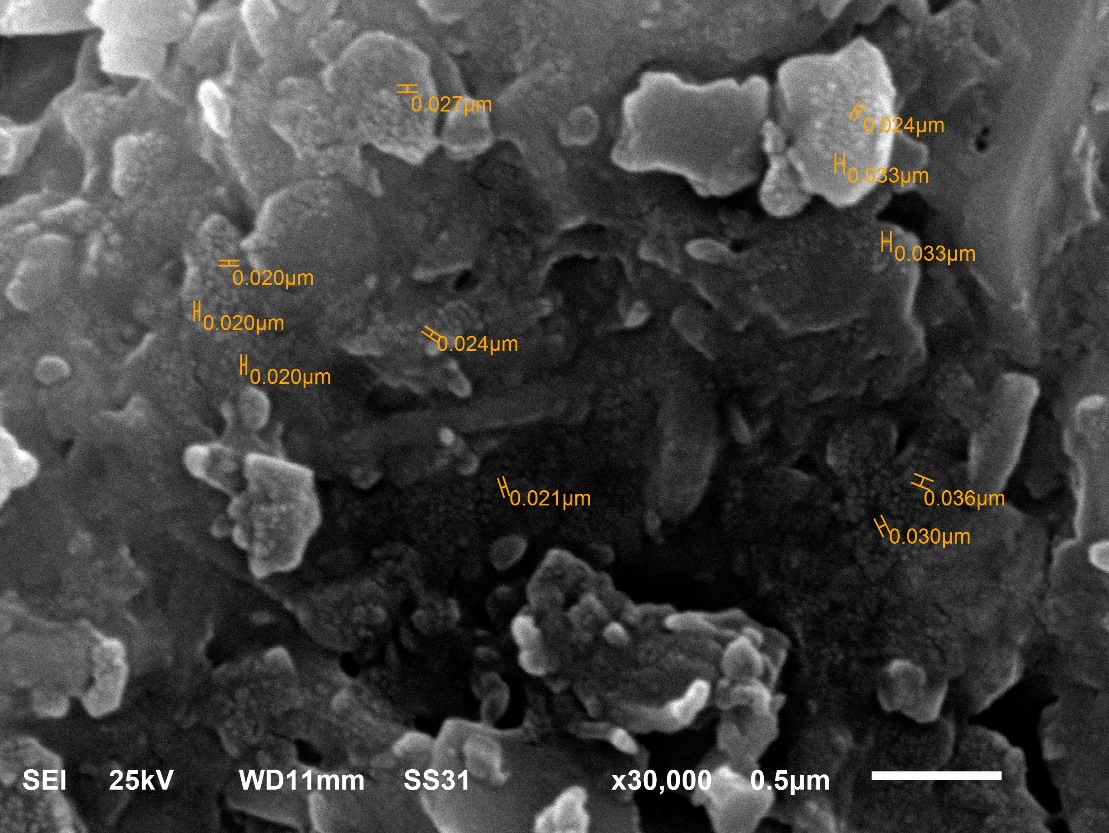


SEM Images for NF-CH-CurNPs nanosystem


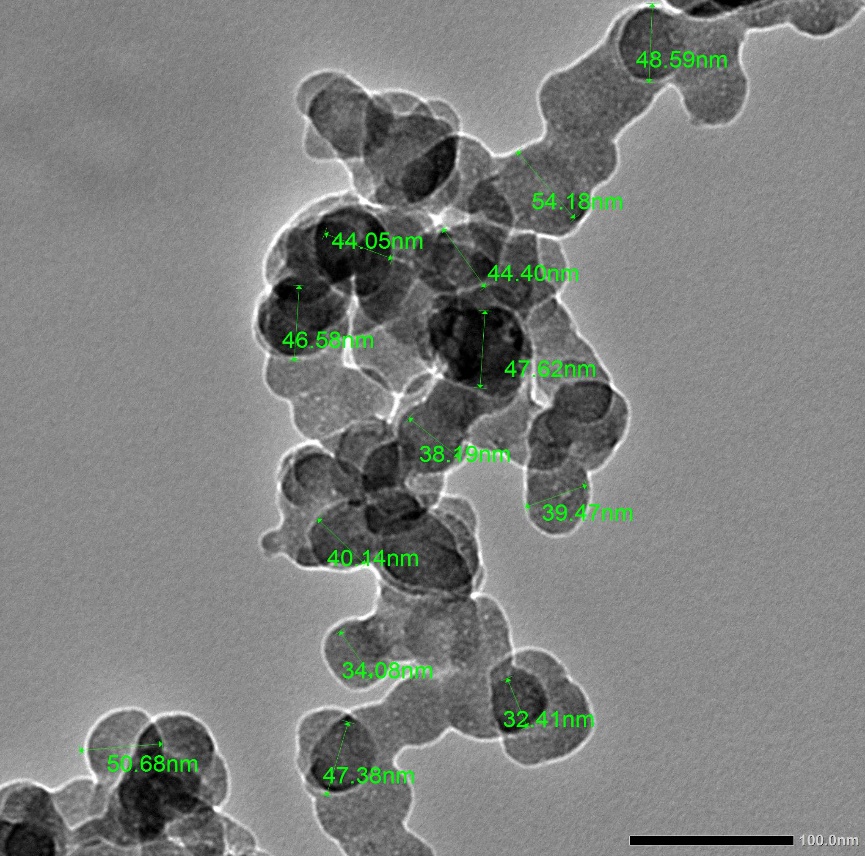


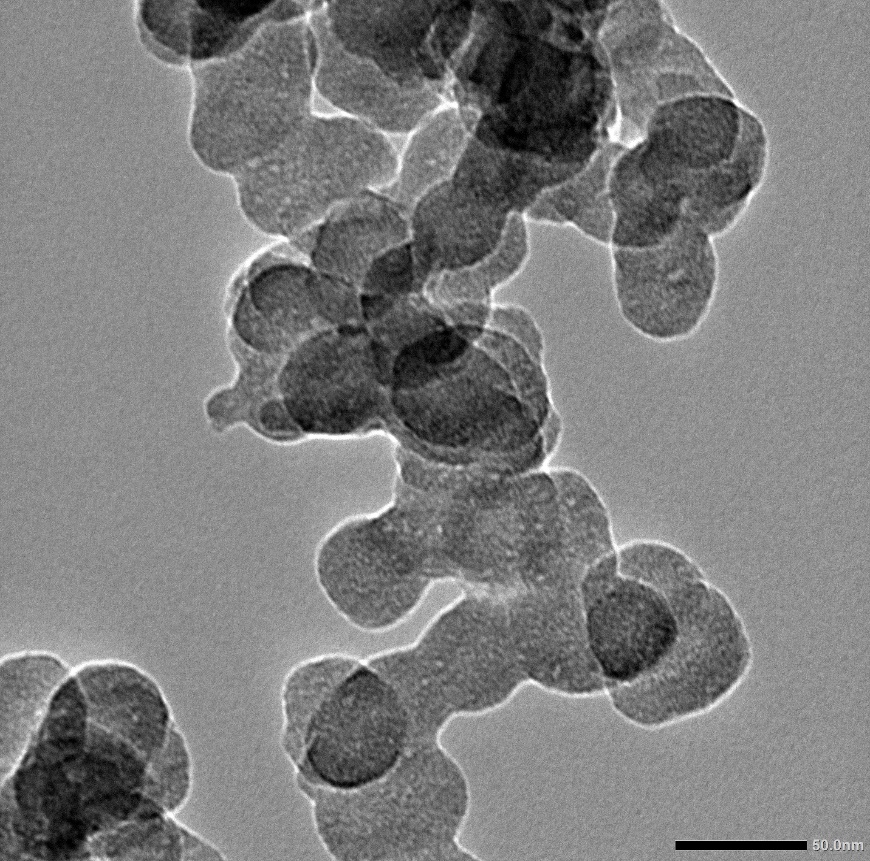


HTEM images for NF-CH-CurNPs nanosystem


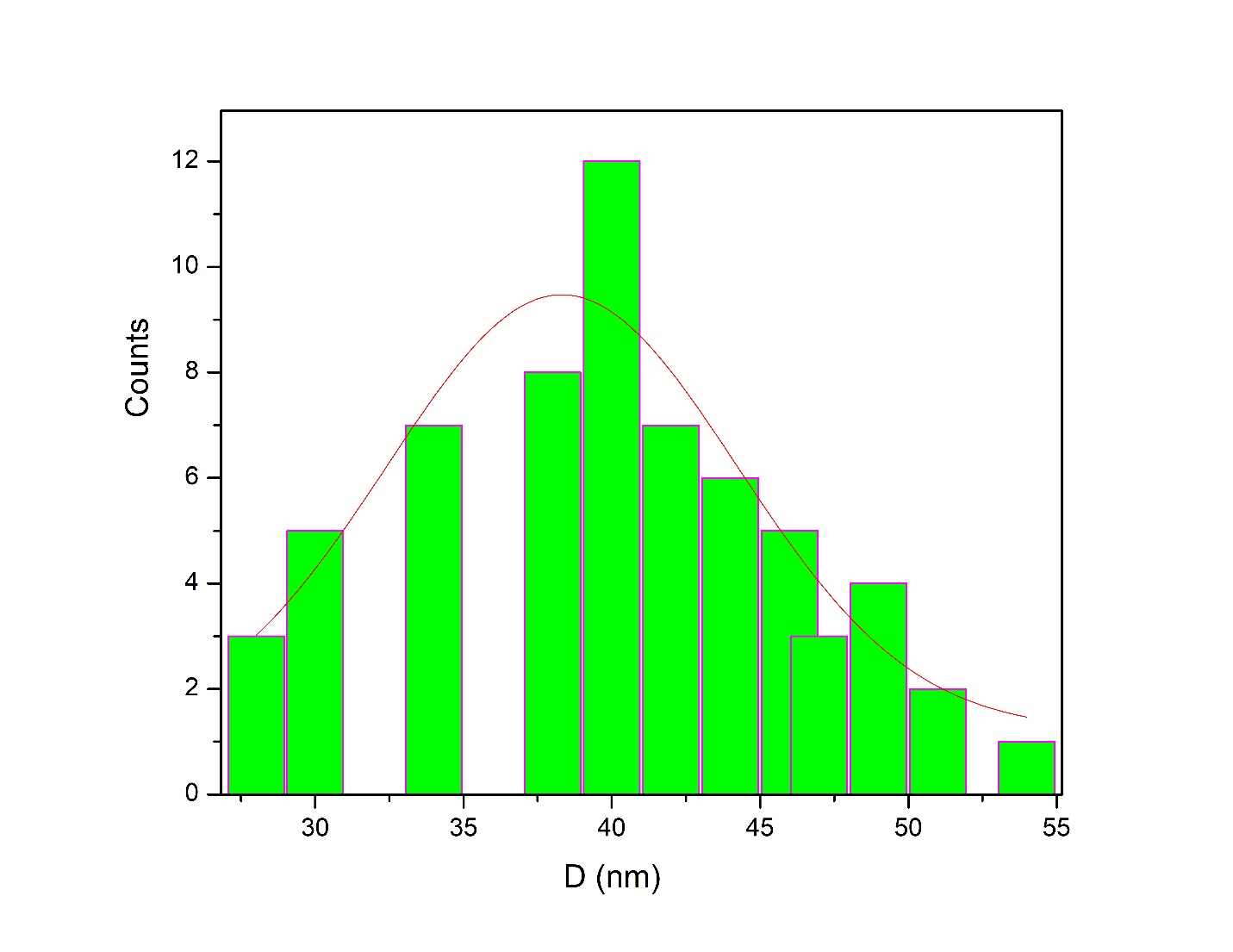


Nanoparticle size distribution obtained from HTEM image for NF-CH-CurNPs nanosystem
